# We are good to grow: dynamic integration of cell wall architecture with the machinery of growth

**DOI:** 10.3389/fpls.2012.00187

**Published:** 2012-08-22

**Authors:** Matheus R. Benatti, Bryan W. Penning, Nicholas C. Carpita, Maureen C. McCann

**Affiliations:** ^1^Department of Biological Sciences, Purdue UniversityWest Lafayette, IN, USA; ^2^Bindley Bioscience Center, Purdue UniversityWest Lafayette, IN, USA; ^3^Department of Botany and Plant Pathology, Purdue UniversityWest Lafayette, IN, USA

**Keywords:** cell wall, cellulose, dicots, extensibility, grasses, growth, pectin, signaling

## Abstract

Despite differences in cell wall composition between the type I cell walls of dicots and most monocots and the type II walls of commelinid monocots, all flowering plants respond to the same classes of growth regulators in the same tissue-specific way and exhibit the same growth physics. Substantial progress has been made in defining gene families and identifying mutants in cell wall-related genes, but our understanding of the biochemical basis of wall extensibility during growth is still rudimentary. In this review, we highlight insights into the physiological control of cell expansion emerging from genetic functional analyses, mostly in Arabidopsis and other dicots, and a few examples of genes of potential orthologous function in grass species. We discuss examples of cell wall architectural features that impact growth independent of composition, and progress in identifying proteins involved in transduction of growth signals and integrating their outputs in the molecular machinery of wall expansion.

## INTRODUCTION

Cell expansion integrates “loosening” of existing architecture with synthesis and deposition of new wall components. Golgi-associated syntheses of matrix polysaccharides and their subsequent secretion is coordinated closely with cellulose synthesis at the plasma membrane, which is in turn coordinated with dynamic assembly and rearrangement during wall extension (Cosgrove, 2005). Multiple signal pathways regulate wall biophysics to permit cell expansion by controlling expression of cell wall-related genes. For example, the brassinosteroid-activated transcription factor BES1 binds to promoter elements of most cellulose synthase (*CesA*) genes (Xie et al., 2011), and NAC and MYB domain-containing transcriptional factors bind to AC-elements of promoters of genes involved in polysaccharide and monolignol syntheses (Zhao and Dixon, 2011).

In elongating cells, cellulose microfibrils are laid down in helical patterns transverse to the long axis of the cell, and are separated and reoriented by tangential forces generated by turgor. Walls of grasses and those of other flowering plants interlace the microfibrils with different matrix constituents (Carpita and Gibeaut, 1993). All dicots and about one-half of all monocots have type I cell walls: a framework of cellulose microfibrils cross-linked primarily by xyloglucans (XyGs) and embedded in a complex matrix of pectic polysaccharides (McCann and Roberts, 1991; Carpita and Gibeaut, 1993); in type II cell walls of the grasses and other commelinid monocots, cellulose microfibrils are cross-linked primarily with glucuronoarabinoxylans (GAXs). Pectins are a small proportion of the matrix polymers, with GAXs providing most of the negatively charged matrix of the type II cell wall (Carpita, 1996). Structural proteins comprise up to 10% of mass of type I walls, whereas networks of phenylpropanoids are deposited in the type II wall. In contrast to other commelinids, the grasses (Poales) contain a mixed-linkage (1 → 3),(1 → 4)-β-D-glucan that is synthesized during cell expansion of grasses and hydrolyzed when growth ceases (Buckeridge et al., 2004).

Cell elongation results in more subtle changes in cell wall composition and architecture than can be revealed by chemical analyses of whole plant organs. Immuno-labeling experiments using a panel of monoclonal antibodies reveals that unique combinations of epitopes are present in highly nuanced patterns along the Arabidopsis root (Pattathil et al., 2010). Infrared spectra of cell walls of maize coleoptiles show that distinct compositional changes occur at 1.5-day intervals (McCann et al., 2007). This dynamic, cellular heterogeneity may be required for wall-modifying activities to alter biophysical properties at precise developmental stages. Tobacco leaves expressing an inducible cucumber α-expansin (*CsEXP1*) promotes leaf growth maximally at the mid-stage of leaf growth (Sloan et al., 2009).

## ARCHITECTURAL CHANGES IN CELL WALLS IMPACT ANISOTROPIC GROWTH

When the structure of the cell wall is severely compromised, the consequence is a strong inhibition of organ elongation. Mutants of primary wall cellulose synthases *CesA1*, *radial swelling1* (*rsw1*), and *CesA6,* *procuste1 (prc1)*, have reduced anisotropic growth in roots and hypocotyls (Arioli et al., 1998; Fagard et al., 2000). In *prc1*, cellulose content is only reduced by about one-third, but the mutation results in a fourfold inhibition of growth (Fagard et al., 2000). Anisotropic distributions of microfibril angles deposited during the initial growth phase can account for the growth inhibition if the wall is modeled as a composite material (MacKinnon et al., 2006). Reorientation from transverse to longitudinal in *procuste* hypocotyls also results in failure to fill gaps in cellulose deposition in some regions, possibly contributing to a tendency for cells to rupture (Anderson et al., 2010).

Mutations in genes whose functions are associated with delivery or activation of cellulose synthases, and other plasma membrane-resident or membrane-associated proteins, also result in cellulose deficiency and inhibition of elongation growth. *CELLULOSE SYNTHASE INTERACTIVE1 *(*CSI1*) is a microtubule-associated protein that bridges CESA complexes and cortical microtubules, mutation of which affects movement of CESA complexes in the plasma membrane (Gu et al., 2010; Li et al., 2012). Site-directed mutations of the phosphorylation sites of CESA1 to mimic always-on or always-off states provide evidence that CESAs are direct targets of signal pathways impacting elongation (Chen et al., 2010). Mutations in a plasma membrane-associated endo-(1 → 4)-β-D-glucanase, *korrigan* in Arabidopsis (Nicol et al., 1998) and rice (Zhou et al., 2006) or in *COBRA* genes, encoding glycosylphosphatidyl inositol-anchored proteins, are cellulose-deficient and compromised in organ elongation in Arabidopsis (Schindelman et al., 2001; Roudier et al., 2005) and rice (Dai et al., 2011).

Loss of cell wall strength to resist turgor pressure is expected with cellulose deficiencies, but similar swelling phenotypes can occur in mutants unaffected in cellulose synthesis. Mutations in two UDP-sugar interconversion pathway genes encoding UDP-glucose dehydrogenases cause significant reduction of substitutions to the XyG backbone, arabinan side chains of rhamnogalacturonan (RG) I, and the apiose-containing side chains A and B of RG II (Reboul et al., 2011), with phenotypes of swollen and misshapen roots and cotyledons, and shorter hypocotyls and reproductive organs.

Xyloglucan endo-β-transglucosylases/hydrolases (XET/XTHs) catalyze the molecular grafting and/or hydrolysis of XyGs in the primary type I cell wall (Rose et al., 2002; Eklof and Brumer, 2010). Galactose-deficient *mur3* XyGs bind to cellulose *in vivo* and *in vitro* as do wild-type XyGs, but are exceptionally poor substrates for XET – a feature that correlates with cell swelling at the end of growth (Peña et al., 2004). When XyGs of different molecular sizes are fed to pea stems, large polymers of XyG cross-link cellulose microfibrils and slow growth by action of endogenous XET activity, whereas XyG oligosaccharides promote cell elongation (Takeda et al., 2002). RNAi lines with reduced levels of AtXTH18 show decreased primary root growth compared to that of wild-type (Osato et al., 2006), and AtXTH14 and AtXTH26 reduced the extension of heat-inactivated isolated cell walls under constant-load extension (Maris et al., 2009). Also, when growing roots were exposed to either recombinant XTH protein, cell elongation is reduced in a concentration-dependent manner and abnormal root hairs are formed, suggesting a role for XET activity in stiffening of the side-walls of root hairs and cells of the elongation zone.

While XET activities may be associated with maintenance of tensile strength by religating XyGs during growth, the actual stress relaxation of the walls required for growth is induced by expansins (Cosgrove, 2005). The expansin superfamily falls into two major groups, called α- and β-expansin, in both dicots and grasses, but with many more β-expansins in the grasses (Sampedro and Cosgrove, 2005). The α-expansins disrupt hydrogen bonds between polysaccharides (Cosgrove, 2000), including cellulose microfibrils in filter paper (McQueen-Mason and Cosgrove, 1994). The β-expansin clade also contains maize group-1 pollen allergens, which, unlike α-expansins, solubilize homogalacturonans (HGs) and highly arabinose-substituted GAXs from the middle lamellae of maize silks during pollen growth (Tabuchi et al., 2011).

However, modifications to pectins also impact wall mechanical properties in dicots. The *mur1* mutant of Arabidopsis is deficient in GDP-mannose dehydratase activity, resulting in the absence of fucose residues in cell wall polymers of the shoot (Bonin et al., 1997). A slight dwarfism and greatly reduced tensile strength of the floral stem suggested that the fucose-containing side-group of XyG might be important for cross-linking during growth. However, O'Neill et al. (2001) showed that the reduced stem growth and tensile strength of the *mur1* mutant is rescued to near wild-type levels by spraying plants with excess boron, thus promoting the dimerization of fucose-deficient side-chains of RG II. Ryden et al. (2003) showed that the tensile strength of *mur1* etiolated hypocotyls was about half that of wild-type but could be similarly rescued. Rescue of the *mur1* phenotype with boron alone shows that RG II dimers are load-bearing and important for cell and organ growth.

Coordinated cell growth in the context of an organ is far more complex than changing biomechanical properties of individual cell walls, and for which the biophysics of cell layers and volumes control organ form (Green, 1996). Osmotic manipulation of wall tension shows regions of “stiffening” interpreted to provide the mechanical determinants for cell patterning (Kierzkowski et al., 2012). The smooth surface of a meristem gives rise to defined undulations by asymmetric cell expansion in a few cells, and these asymmetries pre-stage the pattern of phyllotaxis (Green et al., 1996). A physical undulation induced by asymmetric application of expansin or induction of its expression is sufficient to induce an entire developmental program of organ development, changing phyllotaxis in the apical meristem or leaf shape (Fleming et al., 1997; Pien et al., 2001).

Pectins also appear to be involved in transduction of biophysical signals. HG is synthesized as a heavily methyl-esterified polymer that are de-esterified by pectin methyl esterases (PMEs) to variable extents during cell elongation. Like expansins, modification of methyl esterification of cell wall pectins is linked to organ initiation and control of the normal pattern of phyllotaxis in the apical meristem (Peaucelle et al., 2008, 2011a). Arabidopsis *PME5* is regulated by the homeodomain transcription factor BELLRINGER; in the *bellringer* mutant, PME5 activity is enhanced in the meristem (Peaucelle et al., 2011b). In contrast, *PME5* expression is down-regulated in the mutant, which results in reduced internode elongation. These data suggest a dual function for *BELLRINGER* – a repressor of* PME* in the meristem dome and an activator of *PME* in the elongating stem.

Hydroxyproline-rich glycoproteins (HRGPs) and glycine-rich proteins (GRPs) become cross-linked in walls at the cessation of growth. However, recent work demonstrates that failure to glycosylate these proteins results in defects early in cell growth. An extensin is required for proper cell plate formation during cytokinesis (Cannon et al., 2008). The Arabidopsis prolyl 4-hydroxylase (AtP4H) hydroxylates prolines of glycoproteins, such as extensins, which are *O*-glycosylated with arabinosyl and galactosyl residues by ER- and Golgi-resident glycosyltransferases (Shpak et al., 1999; Gille et al., 2009), and then cross-linked upon delivery to the cell wall (Held et al., 2004). Further complexity in structure is revealed by expression of “glycomodules” of synthetic extensins, where variations in the extent of hydroxylation and glycosylation are observed in a cell and tissue-specific manner (Estévez et al., 2006). Blocking *O*-glycosylation, either chemically by inhibition of P4H with ethyl-3,4-dihydroxybenzoate or α,α-dipyridyl, or genetically by insertional mutagenesis, result in aberrancies in root hair elongation in Arabidopsis (Velasquez et al., 2011).

## STRUCTURE/FUNCTION RELATIONSHIPS MAY BE DIFFICULT TO UNCOVER BECAUSE OF FEEDBACK/COMPENSATION MECHANISMS IN MUTANT GENOTYPES

While severe defects in wall architecture affect anisotropic growth, there is an enormous plasticity in composition and architecture that may compensate, at least in part, for architectural deficiencies. The cellulose synthesis inhibitor dichlorobenzonitrile induces tomato and tobacco culture cells to synthesize a modified type I wall of more highly cross-linked pectin and protein to replace a cellulose–xyloglucan network, whereas barley cells cross-link GAXs with a more extensive polyphenolic network to make a cellulose-free type II wall (Shedletzky et al., 1992). Arabidopsis cells habituated to grow in the cellulose synthesis inhibitor isoxaben alter their cell walls to compensate for the loss of this structural scaffold, and this response is accompanied by strong up-regulation of a glycine-rich cell-wall protein, the *CELLULOSE SYNTHASE-LIKE D5*, a trichome birefringence-like glycosyltransferase (Bischoff et al., 2010), and a putative glycosyltransferase of unknown function (Manfield et al., 2004). The *mur10* mutation in a secondary wall *CESA7* results in alterations to pectin side-group composition in primary walls in cells surrounding the vasculature of Arabidopsis (Bosca et al., 2006).

In contrast, primary wall cellulose deficiencies resulting from either mutated *CesA3* or *CesA1* result in ectopic lignification (Caño-Delgado et al., 2000, 2003). In both Arabidopsis and rice, mutations in other genes associated with cellulose synthesis, such as *korrigan* and *cobra*, can result in increased pectin content and/or ectopic lignification (Nicol et al., 1998; Sato et al., 2001; Zhou et al., 2006). In the temperature-sensitive mutant allele of *korrigan*, *altered cell wall1*, an increase in pectin content of 62% is observed when cellulose content is reduced by 60% at the restrictive temperature (Sato et al., 2001). In rice, mutation in a *COBRA*-like gene, *Brittle Culm-Like4*, causes a dwarf phenotype with fewer tillers than the wild-type (Dai et al., 2011), and an increase in levels of pectin: several *CesA* and *CslF* genes are up-regulated in the mutant compared to wild-type despite reduced cellulose content, suggesting that interference with cellulose deposition elicits a positive feedback mechanism. Two cellulose-deficient dwarf mutant alleles of *kobito1* (*kob1*), a cell wall-localized protein (Lertpiriyapong and Sung, 2003), have increased pectin content, and an increase in the ectopic deposition of both callose and lignin in dark-grown seedlings (Pagant et al., 2002).

Mutations in a XyG-specific fucosyltransferase (*mur2*) and galactosyltransferase (*mur3*) alter or eliminate the α-L-Fuc-(1 → 2)-β-D-Gal-(1 → 2)-side group, yet the mutant plants are indistinguishable from wild-type (Vanzin et al., 2002; Madson et al., 2003). These mutants compensate for these mutations by enhancing activity of a second galactosyl transferase that adds galactose to the middle xylosyl residue (Peña et al., 2004). Although acid-growth and wall extensibility is reduced, expansins still induce extension growth in the XyG-less *xxt1/xxt2* mutant, even though they are missing the primary target of their activity (Park and Cosgrove, 2012a). While the shoots and floral stem are indistinguishable from wild-type in the *mur3* mutant, tensile strength is reduced in etiolated hypocotyls (Ryden et al., 2003; Peña et al., 2004). The reduced tensile strength was traced to a near absence of galactosyl side-groups on hypocotyl XyG (Peña et al., 2004). Cell growth is similar to wild-type, but the *mur3* hypocotyls present an abnormal swelling and bulging along with an increased diameter of both epidermal and underlying cortical cells.

We have depicted cross-bridging of cellulose microfibrils with XyGs and GAXs as the principal load-bearing interaction in our cell wall models (McCann and Roberts, 1991; Carpita and Gibeaut, 1993). However, double mutations eliminating function of two GT34 xylosyltransferases, *XXT1* and *XXT2*, produce plants with no detectable XyG (Cavalier et al., 2008). Plants grow more slowly, are slightly dwarfed, and form short root hairs with bulging bases, but are otherwise surprisingly healthy. The reduction in XyG content slightly reduces stiffness and tensile strength of *xxt2* and *xxt1xxt2* mutant hypocotyls but does not significantly impact extensibility or organ growth. A third xylosyltransferase mutant, *xxt5*, has a phenotype similar to those observed in *xxt1xxt2* double mutants, and reduced XyG content and xylosylation of the glucan backbone (Zabotina et al., 2008). Extensibility is enhanced several-fold in the *xxt1/xxt2* mutant by treatment of tissues with endoxylanase, polygalacturonase, and other treatments that disrupt matrix polysaccharides other than XyGs, indicating that GAX and HG may functionally replace XyGs (Park and Cosgrove, 2012a). These authors conclude that the inherently high extensibility of the mutant over wild-type indicates a reinforcing role for XyGs as well as being the optimal extensibility determinant for which GAX and HG cannot completely substitute. Taken together, these observations reveal a curious paradox – loss of galactosylation of XyG impacts wall tensile strength in organ failure tests, but complete loss of XyG does not. Park and Cosgrove (2012b) also showed that creep can be induced in wild-type, but not in *xxt1/xxt2* mutant plants, by enzymes active against both cellulose and XyG; a cocktail of XyG-specific and cellulose-specific enzyme activities is not effective. These results suggest that the load-bearing connection between microfibrils and XyGs is in a relatively inaccessible region of interaction rather than the extended regions of XyG that span between microfibrils (Park and Cosgrove, 2012b).

## MEDIATORS OF SIGNAL TRANSDUCTION PATHWAYS AND CELL WALL STATUS HAVE BEEN IDENTIFIED

The *Catharanthus roseus* (Cr) receptor-like kinase (RLK1) family contains 17 Arabidopsis members, of which four, *FERONIA* (*FER*), *THESEUS1* (*THE1*), *HERCULES1* (*HERK1*), and *HERK2*, are implicated in regulation of cell wall deposition and extensibility during growth (Hématy et al., 2008; Steinwand and Kieber, 2010). The *the1 *mutant was identified as a suppressor of the hypocotyl elongation defect observed in the CESA-defective *procuste *(Hématy et al., 2007). No visible change in growth or development is seen in *the1* alone, but combining *the1* mutation with *herk1* or *herk2* results in decreases in petiole length and shoot growth (Guo et al., 2009a,b). The *the1:herk1* double mutant produces a severe dwarf phenotype in the loss-of-function *BRASSINOSTEROID RECEPTOR1* (*bri1*) mutant but partially suppresses the excessive cell elongation phenotype of the gain-of-function mutant of the transcription factor involved in *brassinosteroid response1*, *bes1-D* (Guo et al., 2009b). Mutations in two other Arabidopsis leucine-rich repeat (LRR)-RLKs, *FEI1* and *FEI2*, cause swollen-root phenotypes (Xu et al., 2008).

Matrix polymers other than cellulose are also involved in signal pathways. Extracellular domains of *WALL-ASSOCIATED KINASES* (*WAK*s) directly bind to pectin (Seifert and Blaukopf, 2010; Kohorn and Kohorn, 2012). Mutation of WAK2 or expression of antisense *WAK2* or *WAK4* to reduce levels of WAK proteins results in reduction in cell elongation (Lally et al., 2001; Wagner and Kohorn, 2001; Kohorn et al., 2006). Expression of genes associated with cell wall biogenesis and pathogen response is WAK2-dependent, suggesting a role in relaying pectin-based signals from the cell wall (Kohorn et al., 2009). Arabinogalactan proteins (AGPs) are highly glycosylated extracellular proteins anchored to the plasma membrane without kinase activities. Exogenous AGP induces somatic embryogenesis in carrot cells (van Hengel et al., 2001), and apical cell elongation in moss depends on a functional AGP (Lee et al., 2005). The Arabidopsis *fasciclin-like arabinogalactan1* (*fla*1) mutant reduces shoot formation in callus regeneration (Johnson et al., 2011), and mutation of an *AtAGP19* results in reduction of hypocotyl elongation, but, unlike mutations in structural elements, the reduced growth does not result from changes in numbers of cells, cell width, numbers of layers or hypocotyl diameter (Yang et al., 2007).

## PROSPECTS

Despite differences in the structural components of plant cell walls of dicots and grasses, the architectural principles of their construction are similar, giving rise to biophysical properties that underpin common mechanisms of growth. Perturbations to wall architecture, by altering cellulose synthesis and orientation, cross-linking glycan substitution or methyl esterification of the pectin matrix, reveal sensing mechanisms that result in feedback to other biosynthetic pathways. While some candidate components of the sensing mechanisms are receptor kinases or arabinogalactan proteins, a major challenge will be untangling the direct responses of cells to signal transduction mechanisms from the many indirect effects of a life-long deficiency in mutant genotypes. Establishing systems for inducible timing of wall perturbations, introduced by interference with wall synthesis or signal pathways, is a promising approach to unravel the direct integration of growth signals, wall architecture, and biophysical mechanisms of growth.

## Conflict of Interest Statement

The authors declare that the research was conducted in the absence of any commercial or financial relationships that could be construed as a potential conflict of interest.
